# The Electric Light

**Published:** 1887-01

**Authors:** Charles Dawbarn


### THE ELECTRIC LIGHT.

BY CHARLES DAWBARN.

The nineteenth century has crowned science as king, and the royal edict has gone forth proclaiming that the process of nature is ever from cause to effect. But if there be no "chance," it follows that all this matter of which our globe is built no more gathered itself together, than atoms gather themselves together to make the picture which wins our admiration as it stands upon the artist's easel.

There was force at work, and something more than force, which man mortal calls "intelligence," that first gathered the atoms from the vast realm of space, where perchance they had rested under some wondrous law of separation, and placed them so that the law of attraction and repulsion could begin its mighty work. And, obedient to this law, each atom of matter is ever in motion.

Few think of the process by which intelligence calls the lightning to its service, and compels it to sing the song of science in a rythm that is heard all over the world. But save for this law of atomic motion, man would have neither telegraph, telephone, nor electric light to mould the higher civilization of to-day.

Here is a glass jar moulded by intelligence, though force holds its atoms to that shape. And here also is a metal wire. Its atoms are without voice ; but each moves back and forth in its silent sweep. Yonder are the acids and the metals for the battery, all composed of atoms, but every atom knowing its brother as it dwells in this kingdom of force. Now the battery stands complete, and the force of motion in the acid atom, added to atomic motion in the mineral, startles every atom in the copper wire into an energy that demands a wider sweep for its vibration. Almost in a moment the movement has sped till it is arrested by a break in the wire.

Intelligence has started force upon a journey with a through ticket in his pocket, although he will stop at way stations if suitable arrangements are made for his reception. Here at the break in the wire is fastened a piece of carbon, whose atoms are more sedate in their movements, and know nothing of the giddy frolic of the atom in the copper wire. The force that was in motion is by so much arrested, and glows out as heat; for heat is always arrested motion. But it is the same force, and around that carbon is the atmosphere composed of other atoms that speak in a yet different tongue to that "heat force;" or in other words, compel that "arrested motion" to sparkle out into the darkness as the electric light of to-day.

But it is just the same force that inheres to every atom and to the whole universe of matter. That Electric Light is a child of Intelligence wedded to Force. This divine couple has many children, but there is neither oldest nor youngest born into the realm of Nature, though man claims "life" as the first born, and the electric light as the infant of the nineteenth century.

